# Clinical Outcome of HIV-Infected Patients with Sustained Virologic Response to Antiretroviral Therapy: Long-Term Follow-Up of a Multicenter Cohort

**DOI:** 10.1371/journal.pone.0000089

**Published:** 2006-12-20

**Authors:** Félix Gutierrez, Sergio Padilla, Mar Masiá, José A. Iribarren, Santiago Moreno, Pompeyo Viciana, Leopoldo Muñoz, José L. Gómez Sirvent, Francesc Vidal, José López-Aldeguer, José R. Blanco, Manuel Leal, María Angeles Rodríguez-Arenas, Santiago Perez Hoyos

**Affiliations:** 1 Unidad de Enfermedades Infecciosas, Hospital General Universitario de Elche, Universidad Miguel Hernández Alicante, Spain; 2 Unidad de Enfermedades Infecciosas, Hospital de Donostia San Sebastián, Spain; 3 Servicio de Enfermedades Infecciosas, Hospital Ramón y Cajal Madrid, Spain; 4 Servicio de Enfermedades Infecciosas, Hospital Universitario Virgen del Rocío Sevilla, Spain; 5 Unidad de Enfermedades Infecciosas, Hospital Universitario San Cecilio Granada, Spain; 6 Unidad de Enfermedades Infecciosas, Hospital Universitario de Canarias Santa Cruz de Tenerife, Spain; 7 Unidad de Enfermedades Infecciosas, Hospital Universitari de Tarragona Joan XXIII, Universitat Rovira y Virgili Tarragona, Spain; 8 Unidad de Enfermedades Infecciosas, Hospital La Fe Valencia, Spain; 9 Área de Enfermedades Infecciosas, Hospital de La Rioja, Logroño Logroo, La Rioja, Spain; 10 Departmento de Salud Pública, Universidad Miguel Hernández Alicante, Spain; 11 EVES (Escuela Valenciana de Estudios en Salud) Valencia, Spain; AIDS Research Center, China

## Abstract

**Background:**

Limited information exists on long-term prognosis of patients with sustained virologic response to antiretroviral therapy. We aimed to assess predictors of unfavorable clinical outcome in patients who maintain viral suppression with HAART.

**Methods:**

Using data collected from ten clinic-based cohorts in Spain, we selected all antiretroviral-naive adults who initiated HAART and maintained plasma HIV-1 RNA levels <500 copies/mL throughout follow-up. Factors associated with disease progression were determined by Cox proportional-hazards models.

**Results:**

Of 2,613 patients who started HAART, 757 fulfilled the inclusion criteria. 61% of them initiated a protease inhibitor-based HAART regimen, 29.7% a nonnucleoside reverse-transcriptase inhibitor-based regimen, and 7.8% a triple-nucleoside regimen. During 2,556 person-years of follow-up, 22 (2.9%) patients died (mortality rate 0.86 per 100 person-years), and 40 (5.3%) died or developed a new AIDS-defining event. The most common causes of death were neoplasias and liver failure. Mortality was independently associated with a CD4-T cell response <50 cells/L after 12 months of HAART (adjusted hazard ratio [AHR], 4.26 [95% confidence interval {CI}, 1.68–10.83]; *P* = .002), and age at initiation of HAART (AHR, 1.06 per year; 95% CI, 1.02–1.09; P = .001). Initial antiretroviral regimen chosen was not associated with different risk of clinical progression.

**Conclusions:**

Patients with sustained virologic response on HAART have a low mortality rate over time. Long-term outcome of these patients is driven by immunologic response at the end of the first year of therapy and age at the time of HAART initiation, but not by the initial antiretroviral regimen selected.

## Introduction

In the majority of drug-naive patients, initiation of highly active antiretroviral therapy (HAART) results in a reduction in plasma virus load (pVL) to below the detection limit of currently available assays [1]. Plasma viremia is a strong prognostic indicator of HIV disease progression [2]–[5], and suppression of pVL as much as possible for as long as possible has become the primary aim of antiretroviral therapy at present [6]. Long-term suppression of virus replication is expected to ensure recovery of CD4 T-cell numbers enough to protect against opportunistic infections and hence continuous treatment benefit. While several cohort studies have confirmed the overall long-term effectiveness of potent antiretroviral therapy [7]–[11], limited information exists on clinical outcomes and predictors of disease progression in the setting of constant pVL suppression, an scenario that is becoming increasingly common in HIV medicine.

In the present study, we describe clinical outcomes and analyze predictors of disease progression in patients with sustained viral suppression from a large multicenter observational cohort constituted after HAART availability.

## Methods

### Study population and definitions

This project is a joint activity of a recently created Research Network of Excellence (AIDS research network, RIS) funded by the Spanish Research Council which incorporates basic scientists, immunologists, virologists, clinicians, epidemiologists and statisticians. A total of 10 HIV clinic-based cohorts from 7 of the 17 Autonomous Communities of the country (see appendix) participate in the project. Data were collected during the course of clinical care of HIV patients at cohort sites from January 1, 1997 through December 31, 2003. The cohort characteristics have been previously described in detail elsewhere [12]. Each center provided a set of pre-defined variables in a convertible electronic format. The set of variables included the following: gender, date of birth, most probable route of HIV infection, date of first confirmed HIV positive result in the recruiting centre, AIDS defining conditions (initial and during follow-up) and their diagnosis dates, age at HAART initiation, antiretroviral drugs and regimens prescribed and dates of initiation and finalization, CD4 cell count and pVL values in successive visits as well as dates of measurement, vital status, and date of death. CD4 cell count and pVL measurements were performed by the clinical laboratories associated with each of the participating sites. CD4 cell counts were assessed by flow cytometry. Plasma HIV RNA levels were measured by a commercial quantitative PCR technique (Amplicor HIV [Roche Diagnostic Systems], or branched-chain DNA amplification assay [Chiron Diagnostics]). With the data obtained we defined a cohort of patients over 18 years of age that had at least 6 months of follow-up. Censoring date for the analyses was December 31, 2003. Data were cross-checked with the National AIDS Registry in September 2004. Losses to follow-up were calculated by estimating the percentage of subjects still alive, which had not been seen in the clinics in the 12 months previous to December 31, 2003.

For the purpose of these analyses, we selected all antiretroviral-naive patients who began HAART (i.e., any combination of 3 antiretroviral drugs, including at least 1 protease inhibitor [PI], 1 nonnucleoside reverse-transcriptase inhibitor [NNRTI], or abacavir) since January 1, 1997. Patients were included in the analyses if they met all the following criteria: 1) pre-HAART CD4 cell count of <350 cells/µL and plasma virus load >1000 copies/mL, 2) ≥6 months of follow-up and at least one determination of CD4 cell count and pVL after HAART initiation, 3) a complete virologic response to therapy, defined as maintained virus suppression (pVL below 500 HIV-1 RNA copies/mL) throughout follow-up, and 4) a pVL confirming virologic suppression (<500 HIV-1 RNA copies/mL) within 6 months of the last database update. This value of 500 HIV-1 RNA copies/mL was chosen to overcome the heterogeneity of the assay detection limits used to quantify plasma HIV RNA during the study period in the different medical centres.

The last available CD4 cell count obtained within the 2 months before the start of HAART was termed the pre-HAART CD4 cell count. CD4-T cell responses were defined according to changes in CD4 cell count from the pre-HAART value to 6 months (±2 months) and 12 months (±2 months) after HAART initiation. A “low” CD4-T cell response to HAART was defined as an increase in CD4 cell count <50 cells/µL from the pre-HAART assessment. This cut-off has been previously used in other studies [13]–[16]. AIDS-defining events (ADE) were defined according to the 1993 revised clinical definition of the Centers for Disease Control and Prevention. Reported deaths were investigated for their cause by review of the medical chart.

### Statistical analyses

Outcome measures were deaths and new ADE (i.e. CDC stage C) that occurred after the first 6 months of HAART. Patients who developed a new AIDS disease during follow-up were included only once; recurrences were not considered. The primary endpoint was all cause mortality. We also conducted sub-analyses of nonaccidental mortality (excluding deaths attributable to trauma), and of progression to a new ADE or nonaccidental death.

Univariate analyses were performed by using Kaplan-Meier estimates, and differences between curves were tested by using the log-rank tests. For multivariate analysis, we used Cox proportional-hazards models to determine the factors associated with outcome measures. age, gender, category of transmission, previous AIDS diagnosis, pre-therapy CD4 cell count, pre-therapy pVL, type of HAART combination and CD4-T cell responses to HAART, as defined above, were evaluated as potential predictors of clinical outcome. Age at HAART initiation was analysed as a continuous variable and dichotomized into two groups (<50 years and ≥50 years). This age cut-off is commonly used in other studies [17], [18]. HAART regimens were classified as follows: non-boosted PI-based (a combination of nucleoside reverse transcriptase inhibitors [NRTI] plus any of the following PI: nelfinavir, indinavir, saquinavir or ritonavir), boosted PI-based (a combination of NRTI plus any ritonavir boosted PI), NNRTI-based (a combination of NRTI plus either nevirapine or efavirenz), and nucleoside/nucleotide-based regimens (a combination of 3 or more nucleoside/nucleotides). For the purposes of this analysis we followed the “intent-to-continue-treatment” approach and thus ignored subsequent changes of treatment, including treatment interruptions and terminations. All variables with a *P*<0.1 in the univariate analysis were tried to enter simultaneously in a multivariate Cox model. Patients not known to be dead by December 31, 2003 were censored on the date they were last known to be alive. SPSS (version 11) was used for data management and statistical analysis. A two-tailed *P* value of 0.05 or less was considered significant.

## Results

### Patients characteristics at baseline

The cohort database contained data on 4643 patients with at least 6 months of follow-up. Of these, 73% were men and the median age was 35 (interquartile range [IQR], 31–39) years. Overall, 56.4% were intravenous drug users, 22.6% heterosexuals and 14.4% men who had sex with men. Twenty-one percent were diagnosed of AIDS prior to study entry. The incidence rate of AIDS during follow-up was 3.6 per 100 person-years. Overall, 310 (6.7%) patients died, being the death rate 1.7 per 100 person-years. Twenty-seven percent had started HAART prior to study entry. Of the 3421 treatment-naive subjects, 2613 started HAART during follow-up.

A total of 757 patients fulfilled the inclusion criteria and they were, therefore, eligible for the present analyses. Baseline demographic and clinical characteristics of the patient sample are shown in table 1.

**Table 1:**
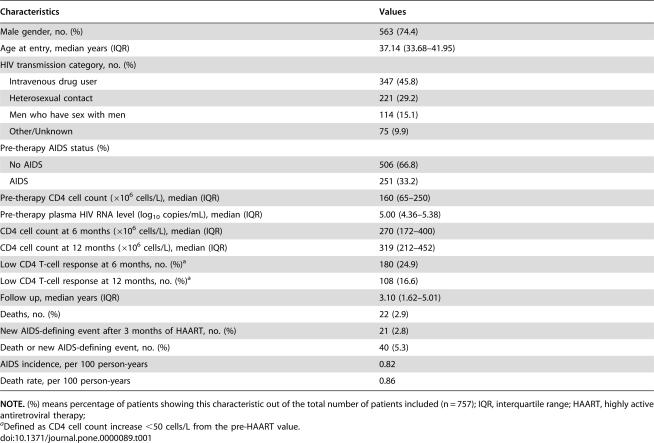
Demographic and clinical characteristics of patients included in the analysis (n = 757).

Characteristics	Values
Male gender, no. (%)	563 (74.4)
Age at entry, median years (IQR)	37.14 (33.68–41.95)
HIV transmission category, no. (%)	
Intravenous drug user	347 (45.8)
Heterosexual contact	221 (29.2)
Men who have sex with men	114 (15.1)
Other/Unknown	75 (9.9)
Pre-therapy AIDS status (%)	
No AIDS	506 (66.8)
AIDS	251 (33.2)
Pre-therapy CD4 cell count (×10^6^ cells/L), median (IQR)	160 (65–250)
Pre-therapy plasma HIV RNA level (log_10_ copies/mL), median (IQR)	5.00 (4.36–5.38)
CD4 cell count at 6 months (×10^6^ cells/L), median (IQR)	270 (172–400)
CD4 cell count at 12 months (×10^6^ cells/L), median (IQR)	319 (212–452)
Low CD4 T-cell response at 6 months, no. (%)^a^	180 (24.9)
Low CD4 T-cell response at 12 months, no. (%)^a^	108 (16.6)
Follow up, median years (IQR)	3.10 (1.62–5.01)
Deaths, no. (%)	22 (2.9)
New AIDS-defining event after 3 months of HAART, no. (%)	21 (2.8)
Death or new AIDS-defining event, no. (%)	40 (5.3)
AIDS incidence, per 100 person-years	0.82
Death rate, per 100 person-years	0.86

**NOTE.** (%) means percentage of patients showing this characteristic out of the total number of patients included (n = 757); IQR, interquartile range; HAART, highly active antiretroviral therapy;

a Defined as CD4 cell count increase <50 cells/L from the pre-HAART value.

### Antiretroviral therapy, CD4-T cell responses and outcome

Antiretroviral therapy administered is detailed in table 2. Sixty one percent of the patients initiated a PI-based HAART regimen (45.3% a non-boosted PI regimen and 15.9% a boosted PI regimen), and 29.7% initiated a NNRTI-based HAART regimen. Only 59 patients (7.8%) started a triple-nucleoside regimen.

**Table 2:**
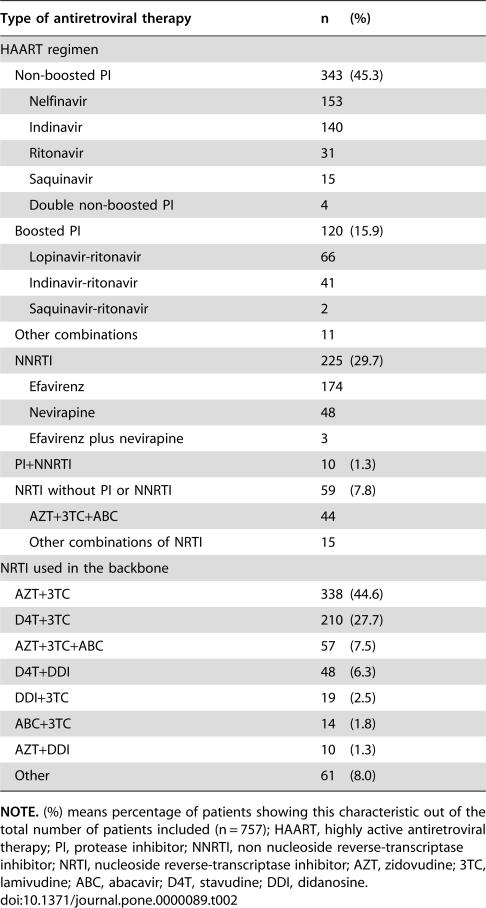
Antiretroviral therapy administered to patients included in the analysis (n = 757).

Type of antiretroviral therapy	n	(%)
HAART regimen		
Non-boosted PI	343	(45.3)
Nelfinavir	153	
Indinavir	140	
Ritonavir	31	
Saquinavir	15	
Double non-boosted PI	4	
Boosted PI	120	(15.9)
Lopinavir-ritonavir	66	
Indinavir-ritonavir	41	
Saquinavir-ritonavir	2	
Other combinations	11	
NNRTI	225	(29.7)
Efavirenz	174	
Nevirapine	48	
Efavirenz plus nevirapine	3	
PI+NNRTI	10	(1.3)
NRTI without PI or NNRTI	59	(7.8)
AZT+3TC+ABC	44	
Other combinations of NRTI	15	
NRTI used in the backbone		
AZT+3TC	338	(44.6)
D4T+3TC	210	(27.7)
AZT+3TC+ABC	57	(7.5)
D4T+DDI	48	(6.3)
DDI+3TC	19	(2.5)
ABC+3TC	14	(1.8)
AZT+DDI	10	(1.3)
Other	61	(8.0)

**NOTE.** (%) means percentage of patients showing this characteristic out of the total number of patients included (n = 757); HAART, highly active antiretroviral therapy; PI, protease inhibitor; NNRTI, non nucleoside reverse-transcriptase inhibitor; NRTI, nucleoside reverse-transcriptase inhibitor; AZT, zidovudine; 3TC, lamivudine; ABC, abacavir; D4T, stavudine; DDI, didanosine.

Median follow up period was 3.1 years (IQR, 1.62–5.01). A total of 63 (8.3%) subjects were lost to follow-up. Lost to follow-up patients were younger (median age [IQR], 34.86 [31.08–38.21] versus 37.26 [33.95–42.49] years; *P*<0.000), and were more likely to be intravenous drug users (62.3% versus 47.7%; *P* = 0.029), but did not differ with respect to baseline pVL, baseline CD4 cell counts, prevalence of AIDS at baseline, or immunological responses at 6 and 12 months of HAART initiation.

CD4- T cells increased in the entire patient sample from a mean of 162 (SD, 103) cells/µL before starting HAART, to 302 (SD, 176) cells/µL and 346 (SD, 176) cells/µL, at 6 and 12 months, respectively, after the initiation of HAART (*P*<0.001). According to previous definition, 180 (24.9%) of the 722 patients with CD4 cell counts available at 6 months had a “low” CD4-T cell response. At 12 months, data were available for 650 (85.9%) patients, with 108 (16.6%) showing a “low” CD4-T cell response

During 2556 person-years of follow-up, 22 (2.9%) patients died (mortality rate 0.86 per 100 person-years), 21 (95.4%) of them were nonaccidental deaths. A total of 21 (2.8%) subjects experienced a new ADE, and 40 (5.3%) developed an ADE or death throughout follow-up. Of the 21 nonaccidental deaths, analysis of cause-specific mortality indicated that 7 (33.3%) patients died of neoplasias [lymphoma, 4 cases; non-AIDS-defining cancers (lung 1, pancreas 1, colon 1), 3 cases]; 4 (19%) patients died of liver failure and/or cirrhosis; 4 (19%) of opportunistic or bacterial infections (pneumonia or sepsis 2, disseminated tuberculosis 1, progressive multifocal leukoencephalopathy 1), 1 (4.8%) case from progressive end-organ disease (cardiac and renal), and 1 case committed suicide. Despite a thorough review, cause of death could not be ascertained for the remaining 4 (19%) individuals who died away from the hospital; in all of them the death was sudden and unexpected.

### Predictors of clinical outcome

Cox models were constructed to investigate factors associated with death and development of an ADE after 6 months of HAART initiation (Table 3). Factors associated with death in the univariate model were age at HAART initiation (hazard ratio [HR], 1.06 per year; 95% confidence interval {CI}, 1.02–1.09), and low CD4-T cell response at 12 month of HAART initiation (HR, 4.81; 95% CI, 1.77–11.36). In the multivariate models, both factors remained associated with disease progression. When age was entered in the model dichotomized into two groups (<50 years and ≥50 years), age ≥50 years at HAART initiation (adjusted hazard ratio [AHR], 3.19, 95% CI; 1.13–09.01; *P* = 0.029), and low CD4-T cell response at 12 month (AHR, 4.26; 95% CI, 1.68–10.83; *P* = 0.002) were independently associated with mortality (Table 3).

**Table 3:**
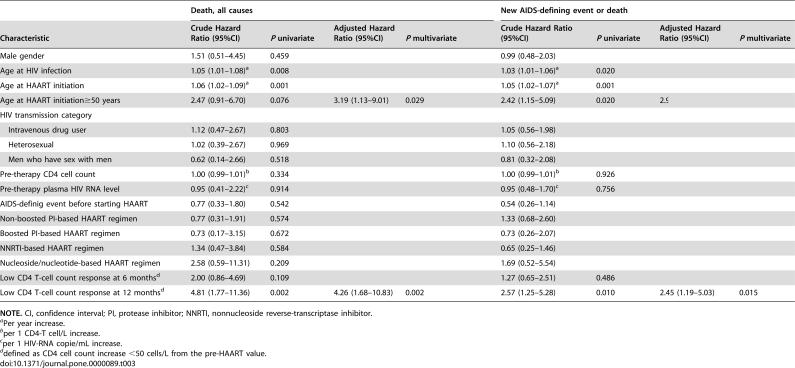
Cox proportional hazards analyses of factors associated with death from all causes, and development of new AIDS-defining events or death, in patients with sustained virologic response to highly active antiretroviral therapy (n = 757).

	Death, all causes				New AIDS-defining event or death			
Characteristic	Crude Hazard Ratio (95%CI)	*P* univariate	Adjusted Hazard Ratio (95%CI)	*P* multivariate	Crude Hazard Ratio (95%CI)	*P* univariate	Adjusted Hazard Ratio (95%CI)	*P* multivariate
Male gender	1.51 (0.51–4.45)	0.459			0.99 (0.48–2.03)	0.978		
Age at HIV infection	1.05 (1.01–1.08)^a^	0.008			1.03 (1.01–1.06)^a^	0.020		
Age at HAART initiation	1.06 (1.02–1.09)^a^	0.001			1.05 (1.02–1.07)^a^	0.001		
Age at HAART initiation≥50 years	2.47 (0.91–6.70)	0.076	3.19 (1.13–9.01)	0.029	2.42 (1.15–5.09)	0.020	2.94 (1.37–6.33)	0.006
HIV transmission category								
Intravenous drug user	1.12 (0.47–2.67)	0.803			1.05 (0.56–1.98)	0.874		
Heterosexual	1.02 (0.39–2.67)	0.969			1.10 (0.56–2.18)	0.781		
Men who have sex with men	0.62 (0.14–2.66)	0.518			0.81 (0.32–2.08)	0.665		
Pre-therapy CD4 cell count	1.00 (0.99–1.01)^b^	0.334			1.00 (0.99–1.01)^b^	0.926		
Pre-therapy plasma HIV RNA level	0.95 (0.41–2.22)^c^	0.914			0.95 (0.48–1.70)^c^	0.756		
AIDS-definig event before starting HAART	0.77 (0.33–1.80)	0.542			0.54 (0.26–1.14)	0.105		
Non-boosted PI-based HAART regimen	0.77 (0.31–1.91)	0.574			1.33 (0.68–2.60)	0.411		
Boosted PI-based HAART regimen	0.73 (0.17–3.15)	0.672			0.73 (0.26–2.07)	0.558		
NNRTI-based HAART regimen	1.34 (0.47–3.84)	0.584			0.65 (0.25–1.46)	0.265		
Nucleoside/nucleotide-based HAART regimen	2.58 (0.59–11.31)	0.209			1.69 (0.52–5.54)	0.387		
Low CD4 T-cell count response at 6 months^d^	2.00 (0.86–4.69)	0.109			1.27 (0.65–2.51)	0.486		
Low CD4 T-cell count response at 12 months^d^	4.81 (1.77–11.36)	0.002	4.26 (1.68–10.83)	0.002	2.57 (1.25–5.28)	0.010	2.45 (1.19–5.03)	0.015

**NOTE.** CI, confidence interval; PI, protease inhibitor; NNRTI, nonnucleoside reverse-transcriptase inhibitor.

a Per year increase.

b per 1 CD4-T cell/L increase.

c per 1 HIV-RNA copie/mL increase.

d defined as CD4 cell count increase <50 cells/L from the pre-HAART value.

When new ADE or death was modelled as the outcome, the 2 same factors remained associated in the multivariate analysis. When age was entered in the model dichotomized, age ≥50 years (AHR, 2.94, 95% CI; 1.37–6.33; *P* = 0.006), and low CD4-T cell response at 12 month (AHR, 2.45; 95% CI, 1.19–5.03; *P* = 0.015) were independent predictors of new ADE or death (Table 3).

The results were unchanged when either nonaccidental mortality or new ADE or nonaccidental mortality were modelled as the outcome (data not shown).

**Figure 1:**
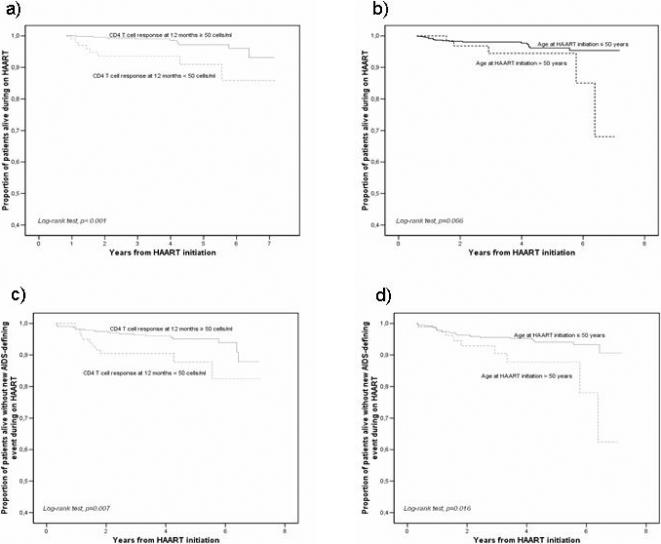
Kaplan-Meier curves of progression in patients with sustained virologic response to HAART, according to CD4-T cell response and age at HAART initiation. (a) Progression to death according to CD4-T cell response; (b) progression to death according to age at HAART initiation; (c) progression to death or new AIDS-defining event according to CD4-T cell response, and (d) progression to death or new AIDS-defining event according to age at HAART initiation.

At Kaplan–Meier estimates, a significant difference was found in terms of proportion of patients experiencing death, and new AIDS defining event or death, during the follow-up period according to CD4-T cell response, and age at HAART initiation (<50 years versus ≥50 years) (Figure 1).

## Discussion

The study has confirmed that patients with sustained virologic response to HAART have a very low mortality rate over time. The analyses have shown that low CD4-T cell responses at 12 months and age at the initiation of antiretroviral therapy are the main determinants of unfavourable long-term outcome. The initial antiretroviral regimen chosen, however, was not associated with different risk of clinical progression. Our results are based on a large population of patients with over 2500 patient-years of observation, which allowed us to examine clinical progression up to more than 3 years after starting HAART. The cohort includes many clinics treating HIV-1-infected patients from different regions of Spain, with a broad spectrum of patient characteristics and antiretroviral regimens. The results should therefore be applicable to many individuals with HIV-1 infection beginning HAART in Spain and elsewhere.

Low immunological response to antiretroviral therapy was the strongest predictor of mortality. This finding is in agreement with those from previous studies assessing clinical outcomes in patients receiving HAART, that have suggested poorer prognosis in subjects with discordant immunologic and virologic responses [13], [15], [19]–[22]. Most of these studies have investigated responses to HAART at 6–9 months, defined as either absolute values attained [21] or increase in CD4 cell counts of at least 25 cells/µL [22] or 50 cells/µL from baseline [13], [15], [21]. Immunologic responses of 50 cells/µL had previously been correlated with a reduced risk for the development of opportunistic infections [14]. Our results give support to the prognostic significance of this threshold, and are in accordance with recent data by investigators from British Columbia [15] analyzing data from 1527 treatment-naive individuals initiating HAART. In that cohort, failure to gain 50 cells/µL from baseline within 3 to 9 months after initiating HAART was found to be a significant predictor of later mortality in patients with virologic suppression. In our study, however, the response at 6 months was less predictive of clinical outcome than that measured after 12 months of HAART initiation.

In addition to suboptimal immunological response, older age at initiation of HAART was found to predict unfavorable outcome in our study. Most studies have shown that compared with younger patients, patients over 50 generally have a slower immunological response to HAART and experience more rapid clinical progression [17], [23]. However, in our study the detrimental effect of age on progression to a new AIDS defining event or death persisted after adjusting for CD4-T cell response, suggesting that age contributes independently to clinical progression.

Several published randomized clinical trials have shown that different antiretroviral regimens have different capacities to decrease the pVL and raise the CD4 cell count thus leading to different clinical efficacy. In addition, it has been suggested that antiretroviral drugs might have effects on risk of AIDS and/or death, which are not mediated by their effect on HIV-RNA and CD4 cell count [24], [25]. In this cohort of patients with sustained virologic response on HAART, however, the initial antiretroviral regimen chosen was not associated with different risk of clinical progression. This finding is in agreement with the results of a recent analysis from the EuroSIDA observational cohort indicating that AIDS/death rates for given CD4 cell count and pVL categories are similar, independently of HAART regimen [26].

An expected finding from the study was the significant contribution of mortality from non-AIDS-defining illnesses, including cancer and end-stage liver disease. These results corroborate previous findings that indicate that the relative proportion of non-AIDS-related death has increased during the period of HAART [27], and that patients with AIDS are now experiencing growing mortality from causes generally referred to as non-HIV-related [28]–[31]. Although some of these deaths may be the result of a number of factors including common comorbidities that occur in patients with AIDS, a relationship to immunodeficiency can not be ruled out. A recent analysis from the D:A:D cohort has shown that deaths from causes generally supposed to be unrelated to HIV, such as liver-related deaths and deaths from non-AIDS malignancies, were in fact more likely to occur in persons with low CD4 cell counts, indicating that both HIV-related and “non-HIV-related” mortality may be associated with immunodeficiency [32].

A number of limitations in our study should be noted. First, to be included in the analysis, patients recorded in the database must have had at least a CD4 cell count and pVL assessment within 6 to 12 months after starting HAART. As a result, patients who died or were lost to follow-up before a second CD4 cell count assessment were not included, and consequently the risk for clinical progression may have been underestimated. The 8% losses to follow-up could have introduced a number of other different biases into our results. Lost to follow-up patients were younger and they were more likely to be intravenous drug users. However, considering the total number of losses and the lack of differences in other baseline parameters, including immunological responses to HAART, with respect to the other group, the effect of such biases would likely to be small. The analyses of the effect of different antiretroviral regimens is limited by the fact that it was a nonrandomized, observational cohort study, and the selection of therapy depended on the treating clinicians criteria and the availability of the antiretroviral drugs throughout the study. Finally, given the large number of participating sites, variations in CD4 cell counts and pVL might have occurred. A potential impact of such variations on the study results cannot be ruled out.

Several confounding factors might have influenced the results. Unfortunately, hepatitis C virus (HCV) serostatus and adherence to therapy were not included among the set of pre-defined variables in the database, therefore the contribution of these factors to immunological response and clinical outcomes could not be ascertained in the study. The influence of HCV co-infection in immunological response to HAART remains controversial. Recent data from the British Columbia cohort suggest that HCV antibody-positive HIV-infected patients may have an altered immunologic response to HAART [33], a finding that has not been confirmed by EuroSIDA study group [34]. Although adherence to therapy has previously been shown to be an important determinant of mortality in HIV-positive individuals on HAART [35], [36], our study population maintained successful virologic suppression throughout the follow-up, thus minimizing the importance of this factor in our analyses.

Despite the limitations, the study represents some of the first substantial body of data on long-term outcome of patients with sustained virologic response on HAART. The analyses were conduced in patients who started therapy with a broad spectrum of antiretroviral drugs and we selected the CD4 threshold at which antiretroviral therapy is currently recommended [6]. The results should therefore be generalizable to many patients beginning HAART today.

In summary, our results indicate that clinical outcome of patients who maintain a durable virologic suppression on HAART could be driven by the immunologic response at 12 months and the age at the time of HAART initiation, but not by the initial antiretroviral regimen chosen. The fact that the CD4-T cell response is strongly associated with long-term clinical outcome indicates that both immunologic and virologic responses are important when assessing the effectiveness of antiretroviral drugs. The study results suggest the need for alternative strategies to amend immune response in patients with low CD4-T cell increases at the end of the first year of therapy. Older age at initiation of HAART was also found to predict unfavorable outcome, indicating that age at which therapy is started should be carefully considered.
